# Antimicrobial stewardship capacity and infection prevention and control assessment of three health facilities in the Ashanti Region of Ghana

**DOI:** 10.1093/jacamr/dlac034

**Published:** 2022-04-09

**Authors:** Obed Kwabena Offe Amponsah, Alex Owusu-Ofori, Nana Kwame Ayisi-Boateng, Joseph Attakorah, Mercy Naa Aduele Opare-Addo, Kwame Ohene Buabeng

**Affiliations:** 1 Department of Pharmacy Practice, Faculty of Pharmacy and Pharmaceutical Sciences, College of Health Sciences, Kwame Nkrumah University of Science and Technology, Kumasi, Ghana; 2 Department of Microbiology, Komfo Anokye Teaching Hospital, Kumasi, Ghana; 3 School of Medicine and Dentistry, College of Health Sciences, Kwame Nkrumah University of Science and Technology, Kumasi, Ghana; 4 University Hospital, Kwame Nkrumah University of Science and Technology, Kumasi, Ghana

## Abstract

**Background:**

Addressing antimicrobial resistance (AMR) requires the rational use and optimization of available resources for prevention and management of infections. Structures in health facilities to support optimal antimicrobial therapy and AMR containment therefore need assessment and strengthening.

**Objectives:**

To assess antimicrobial stewardship (AMS) capacity and conformance to National and WHO Infection Prevention and Control (IPC) guidelines in three hospitals in Ashanti region of Ghana.

**Methods:**

A cross-sectional study using WHO’s hospital questionnaire for AMS capacity assessment, and Infection Prevention and Control Framework (IPCAF) to assess IPC practices in the three hospitals.

**Results:**

All the facilities had Drug and Therapeutics and IPC Committees with microbiology laboratory services. H3 and H1 did not have a formal AMS programme or an organizational structure for AMS. However, both institutions had a formal procedure to review antibiotics on prescriptions for quality assessment and relevance. H2 and H1 did not participate in any surveillance of antibiotic resistance patterns or consumption. H1 had basic, while H2 and H3 had intermediate-level IPC systems scoring 385, 487.5 and 435.8 out of 800 respectively.

**Conclusions:**

All the facilities assessed had AMS capacity and IPC conformity gaps that require strengthening to optimize antimicrobial use (AMU) and successful implementation of IPC protocols. Regular surveillance of antimicrobial consumption and microbial resistance patterns should be an integral part of activities in health institutions to generate evidence for impactful actions to contain AMR and improve AMU.

## Introduction

The continued spread of antimicrobial resistance (AMR) is a major public health concern globally.^1^ This is driven primarily by the inappropriate use of antibiotics,^2^ inappropriate prescription practices, inadequate patient education, limited diagnostic facilities, unauthorized sale of antimicrobials, lack of appropriate and functioning drug regulatory mechanisms, and non-human use of antimicrobials such as in animal production.^3^

A number of interventions have been employed in the fight against AMR. These include institutional adoption and effective implementation of proven infection prevention and control (IPC) strategies, rational pharmacotherapy and control of infectious diseases. Incentivizing the pharmaceutical industry to develop new antimicrobials against AMR has been another strategy. Ultimately, the solution may be in the setting up and implementation of pragmatic antimicrobial stewardship (AMS) mechanisms to protect available antimicrobials which are still effective against pathogenic microbes, including those that are drug resistant.^4,5^ Antimicrobial stewardship programmes (ASPs) are needed to protect the effective antimicrobials available now and those to be developed in the future.^5–7^ AMS programmes are often interlaced with IPC to better optimize the fight against AMR. This is especially important in dealing with healthcare-associated infections (HAIs) which are often due to resistant pathogens with an estimated 15% burden in low and middle income countries.^8–10^ IPC systems reduce the incidence of infections while AMS ensures optimized drug therapy in infection management, in addition to providing a double-edged sword to fight AMR.^11^

Addressing AMR thus requires various resources that must be applied efficiently to achieve sustainable impact. This may be part of the hindrances encountered in the fight against AMR in the low-and middle-income countries (LMICs).^12,13^ It is therefore important that any resources harnessed to fight against AMR in LMICs should be maximized to achieve the desired impact. This therefore requires in-depth knowledge and analysis of the AMR-related problems and developing local solutions that fit within the global plans against AMR. It is especially useful that the resources available are applied responsibly for impact in low-resource countries in this fight. A national action plan (NAP) for AMR containment in Ghana has been developed in line with the WHO, Food and Agriculture Organisation (FAO) and World Organisation for Animal Health (OIE)’s Global Action Plan, based on the One Health approach.^14^ The strategic objectives of Ghana’s NAP include reducing incidence of infections through IPC policies and practices, as well as optimizing antimicrobial use in infection management. Hospital settings in Ghana are among the key sectors in human health where both strategies need to be well utilized. The WHO has further developed practice manuals on the implementation of the eight WHO Guidelines on core components of IPC programmes at national and at facility levels.^15^ The detailed aspects of the IPC components differ widely between facilities from different income levels and even within individual countries.^16,17^ WHO has therefore released the Infection Prevention and Control Assessment Framework (IPCAF)^18^ which is in the form of a questionnaire to assess IPC structures in individual health facilities. This provides a standardized means of assessing institutional capacity and conformity to IPC recommendations.

To the best of our knowledge, no studies have assessed institutional capacity for AMS alone or concurrently with IPC initiatives in Ghana, and especially in the Ashanti region. A study that assessed IPC initiatives in Ghana assessed five out of eight possible IPC components of the standard tool in the facilities.^19^ Thus, a more comprehensive assessment is needed to ensure all gaps in practice are identified and addressed for health and safety. This will provide guidance on the need for pragmatic interventions through AMS and IPC to address and/or contain the high burden of AMR.^20,21^ Institutions providing health services to patients in Ghana therefore need to be assessed for their capacity to implement such interventions. This study aimed to assess the antimicrobial stewardship capacity in three healthcare facilities in the Ashanti region of Ghana, in addition to assessing their conformance to WHO’s eight core IPC components.

## Methods

### Study design and setting

This was a cross-sectional study, conducted at three facilities coded H1, H2 and H3. These facilities were classified according to the WHO methodology for point prevalence survey (PPS)^22^ used for the AMS capacity assessment. The facilities provide a wide range of services to residents in the Ashanti region of Ghana. Together, they serve thousands of individuals in the urban, peri-urban and rural Ashanti region as well as referral patients from neighbouring regions. They are also key sites for surveillance of antibiotic use and antimicrobial resistance where previous assessments have shown high antibiotic consumption. These sites were thus chosen as they are part of an overarching project to institute AMS and improve antibiotic use in the region.

H1 was classified as a primary hospital, H2 as secondary and H3 as a tertiary facility. Further characteristics of the facilities are summarized in Table 1.

**Table 1.** Characteristics of health facilities

Facility	Geographic location	Catchment population	Number of beds^a^	OPD visits [Admissions]^a^	Facility level	Services provided
H1	Ejisu municipal^23^	143* *762	76	41* *888 [5745]	Primary	Outpatient Services, Surgery & Obstetric, Maternal & Reproductive Health services, General Administration, Eye care Services, Laboratory services, Psychiatry services.
H2	Asante Akim North District^24^	117* *245	250	127* *492 [11* *897]	Secondary	General and specialist care in Internal Medicine, General Surgery, Child Health, Obstetrics/Gynaecology, Ophthalmology, Ear, Nose and Throat, Sickle Cell, Infectious Diseases.
H3	Kumasi Metropolitan^25^	730* *249	120	91* *527 [5218]	Tertiary	General care and specialist care in Internal Medicine, Surgery, Paediatrics, Obstetrics and Gynaecology, Dental care, Mental health, Infectious Diseases, Emergency services, Urology, Haematology, Otolaryngology, Ophthalmology and Neurology.

a Values as of 2019.

### Study participants

Each hospital had a focal person selected after enquiry at each facility to complete the hospital questionnaire and the IPCAF. The questionnaires were administered to these individuals; a principal pharmacist at H1 for the AMS capacity assessment and an IPC focal person for the IPCAF, a nurse administrator at H2 and a specialist physician at H3 for both questionnaires. The participants were chosen because of their key roles in the hospitals allowing them to possess first-hand knowledge to answer the questionnaires. Data was obtained from responses of two different people for the AMS capacity and IPCAF at H1. Data from H2 and H3 were obtained for both assessments from one individual selected at each hospital. The questionnaires were administered and retrieved between November 2019 and February 2020 from the various facilities.

### Hospital questionnaire and IPCAF

Assessment of the AMS capacity of the health facilities was done using the Hospital Questionnaire (see Supplementary data available at*JAC-AMR*Online) of the WHO methodology for PPS on antibiotic use in hospitals Version 1.1.^22^ The questionnaire was used to assess stewardship capacity under Infrastructure, Policy and Practice Monitoring and feedback. The IPCAF at the facility level tool^26^ was also employed to assess the IPC systems of these facilities in eight key areas including components of their IPC programme, guidelines, education and training, surveillance of HAIs as well as monitoring and audits of IPC practices. The IPCAF has a scoring system with which the level of a facility’s IPC is graded as either Inadequate, Basic, Intermediate or Advanced (Table 2).

**Table 2.** IPCAF Score interpretation^26^

Total score (range)	IPC level	Interpretation
0–200	Inadequate	IPC core components implementation is deficient. Significant improvement is required.
201–400	Basic	Some aspects of the IPC core components are in place, but not sufficiently implemented. Further improvement is required.
401–600	Intermediate	Most aspects of the IPC core components are appropriately implemented. The facility should continue to improve the scope and quality of implementation and focus on the development of long-term plans to sustain and further promote the existing IPC programme activities.
601–800	Advanced	The IPC core components are fully implemented according to the WHO recommendations and appropriate to the needs of the facility.

### Data management and analyses

Data was collected from each hospital, appropriately identified and coded accordingly for data entry. All the data obtained were entered into a REDCap® database and exported into STATA™ 14 for analyses.^27,28^ Missing data were entered as ‘NO’ or ‘NOT AVAILABLE’ in the database. Descriptive analysis was performed and presented in a form of tables.

### Ethics

Ethics approval was obtained from the Committee on Human Research, Publications and Ethics of the Kwame Nkrumah University of Science and Technology (KNUST) after obtaining approval to conduct the study from each of the facilities (CHRPE/AP/654/19).

## Results

### WHO Hospital Questionnaire on AMS (AMS capacity assessment)

All but one question relating to the list of stockout antibiotics in the questionnaire were answered in all the facilities. The responses are shown in Tables 2 to 4. All facilities had drug and therapeutics and IPC committees with access to microbiology services. H3 and H1 reported not having a formal ASP or organizational structure for AMS (Table 3).

**Table 3.** Assessment of health facility's infrastructure

Infrastructure	H1	H2	H3
Functioning drugs and therapeutics committee	Yes	Yes	Yes
Functioning IPC committee	Yes	Yes	Yes
Functioning pharmacovigilance committee	Yes	Yes	Yes
Microbiology lab/division in hospital	No	Yes	Yes
Microbiology service available outside hospital	Yes	Yes	Yes
Formal AMS programme	No	Yes	No
Formal organizational structure for AMS	No	Yes	No
Antimicrobial stewardship team available	No	Yes	Yes
Physician leader for AMS activities	No	Yes	No
Responsible pharmacist for rational antibiotic use	Yes	Yes	No
Salary support for time dedicated to AMS	No	Yes	No
IT capability to support AMS activities	Yes	Yes	Yes
Outpatient parenteral antibiotic therapy unit	No	Yes	Yes

**Table 4.** Assessment of policy and practice to support antimicrobial stewardship

Policy and practice	H1	H2	H3
Continuously updated antibiotic formulary	No	Yes	No
Antibiotic formulary based on essential drug list	No	Yes	Yes
Antibiotic guideline available	No	Yes	No
Local antibiotic guideline available in facility	No	Yes	No
Local guidelines based on local susceptibility	No	Yes	Yes
Written policy to document antibiotic indication	Yes	Yes	No
Specific antibiotics need prior approval for use	No	Yes	No
Formal procedure for antibiotic review	Yes	No	Yes

H3 and H1 did not have a continuously updated antibiotic formulary or guideline but had a formal procedure for antibiotic review after prescription. H2 reported ‘Yes’ to all questions except on the formal procedure for review of antibiotic therapy (Table 4).

H3 and H2 reported monitoring antibiotic use while H3 and H1 reported not monitoring antibiotic use by grams of antibiotic per patient per day and by hospital denominator. None of the facilities reported producing a cumulative antibiotic susceptibility report and one reported producing an annual report on AMS in the past year. H2 and H1 do not participate in any national surveillance on antibiotic resistance or on antibiotic use (Table 5).

**Table 5.** Assessment of monitoring and feedback practices of health facility

Monitoring and feedback	H1	H2	H3
Monitor that all antibiotics have documented indications	Yes	Yes	Yes
Audit of surgical antibiotic prophylaxis	No	No	No
Results of antibiotic audits communicated with prescribers	Yes	Yes	No
Facility monitors antibiotic use	No	Yes	Yes
Facility monitors antibiotic use by grams of antibiotic per day	No	Yes	No
Monitored antibiotic use reported by hospital activity denominator	Yes	No	No
Annual report on AMS produced in the last year	Yes	No	No
Cumulative antibiotic susceptibility report produced in past year	No	No	No
Participating in national AMR surveillance programme	No	No	Yes
Participating in national antibiotic use surveillance programme	No	No	Yes

### Infection Prevention and Control Assessment Framework (IPCAF)

The total and subtotal scores of the health facilities as regards their assessment of compliance with the eight core components of an IPC programme are presented. Due to the scoring system of the IPCAF, any unanswered questions, if any, are entered as zero. Table 6 shows the detailed scores of the health facilities. H1 had a Basic IPC level, H2 and H3 both had Intermediate level IPC systems. Overall, the facilities scored lowest in component 5, scoring 38.3%, and highest in component 2, scoring 86.7%.

**Table 6.** IPCAF Health Facility Scores for the various IPC components

Section (Core component)	Subtotals (%)			
	H1	H2	H3	Average
1. IPC programme	55	42.5	55	50.8
2. IPC guidelines	80	87.5	92.5	86.7
3. IPC education and training	70	40	60	56.7
4. HAI surveillance	0	70	55	41.7
5. Multimodal strategies	0	80	35	38.3
6. Monitoring/audits of IPC practices and feedback	72.5	17.5	30	40
7. Workload, staffing and bed occupancy	50	55	30	45
8. Built environment, materials and equipment for IPC at the facility level	57.5	95	77.5	76.7
Final total score	385	487.5	435	435.8
Interpretation	Basic	Intermediate	Intermediate	Intermediate

## Discussion

This study assessed the capacity of the institutions to institute and sustain antimicrobial stewardship as well as conformity to the WHO Core components of IPC in the institutions. The study identified a number of gaps that need to be resolved to ensure optimal antimicrobial use in the hospitals as well as IPC.

### Antimicrobial stewardship assessment

The results of the AMS capacity assessment were similar to those observed from studies in Nigeria, which utilized a similar approach to assess stewardship programmes.^29,30^ On infrastructure, H1 reported not having a microbiology laboratory or division within the hospital. This is important in establishing local microbial resistance data to guide the rational use of antibiotics, an appropriate target for quality improvement in internal capacity for AMS. H1 and H3 did not have a physician leader for AMS nor a pharmacist responsible for ensuring appropriate antibiotic use in H3. In one of the studies from Nigeria,^30^ almost three-quarters of the hospitals surveyed did not have a physician leader for AMS while about four-fifths did not have a pharmacist responsible for ensuring appropriate antimicrobial use. In another study,^29^ five of six facilities had a physician leader as well as a pharmacist involved. These personnel, including, but not limited to physicians, pharmacists and nurses, are needed for the success of AMS as expertise from various fields is needed to sustain a programme. For instance, pharmacist-led and pharmacist-involved ASPs have been shown to improve patient outcomes and antibiotic use even in low-resource settings.^4,31,32^ This may be attributed to Pharmacists’ expertise and knowledge in medicines and their use in clinical practice. A multidisciplinary team is indispensable to the success of AMS programmes implemented in hospitals. The 2017 Geneva IPC Think Tank also appropriately recommends one full-time specifically trained IPC nurse per 250 beds and/or a dedicated physician trained in IPC.^33^

Under policy and practice, H1 and H3 reported not having a facility antibiotic formulary that is updated continuously, an antibiotic guideline or local antibiotic guideline. Formularies are an important resource that aid clinicians and healthcare teams to be able to choose appropriate empirical agents for specific indications. This may also be a target for implementation to improve antibiotic use in the facilities. It was also not routine in the two facilities for specific agents to be approved for use by a physician or pharmacist, compared with 88%^30^ and 44%^29^ of facilities in Nigeria. This could be an important target for stewardship activities in restricting the use of certain agents in the facility due to local susceptibility patterns or international standards.^9^ This may especially be important for the use of antibiotics placed in the *Reserve* category of the WHO *AWaRe* classification.^34,35^

Under monitoring and feedback, a key finding was that none of the facilities reported auditing or reviewing surgical antibiotic prophylaxis choice and duration. This is very low compared with the 24% of facilities that do this in Nigeria.^29,30^ Surgical prophylaxis is among the top indications for antibiotic use in hospitals and is associated with high rates of inappropriate use.^36–38^ It is important therefore that such audits and reviews are carried out to improve appropriate antibiotic prescribing. This may be an important target for improvement since antibiotic use for surgeries and surgical prophylaxis globally, including some developing countries, was between 17.8% to over 54% of all antibiotic indications.^9,39–42^

The results of the AMS capacity assessment are baseline metrics for subsequent implementation of AMS in the hospitals. These findings are important benchmarks that allow objective assessment of the outcomes of any interventions that may be put in place to improve antibiotic prescribing. The findings suggest that there is the need for a scale up of antimicrobial consumption and AMR surveillance in these facilities to be able to make objective decisions to improve their use.

### IPC assessment

No facilities were considered inadequate in IPC systems with respect to the IPCAF, better than observed in a similar study conducted in 56 facilities in Ghana.^19^ That study, however, assessed the facilities on the WHO IPC core components (CC) 1, 2, 3, 7 and 8 whereas this study assessed all eight CCs, providing a potential buffering effect on the scores in this study.^19^ On the average, the facilities performed lowest in CC5 which assesses multimodal strategies for IPC. This is a relatively new concept in IPC, which basically seeks to translate evidence and guidelines recommendations into healthcare practice aimed at changing health worker behaviour.^43,44^ This component was also the lowest scoring in a similar study conducted in Germany, supporting the need to invest in these strategies to improve IPC practices.^45^ This may be achieved through multidisciplinary team cooperating to develop locally appropriate tools and programme bundles that are locally acceptable and feasible to achieve and sustain IPC behaviour change.

The facilities performed highest in CC2 which generally meant that the facilities possessed or had the internal capacity to develop guidelines for IPC structures to function well. This is important when considered in relation to the average scores from the components requiring implementation of those guidelines. For instance, less than half of the possible total score (100) was obtained for CC6, CC4 and CC7. These are on monitoring of IPC practices, HAI surveillance and workload, others are: staffing and bed occupancy, respectively, in ascending order of the scores obtained by the institutions. This highlights the main problem in these facilities as potentially being one of implementation, since high scores in CC2 imply the facilities do not lack IPC guidelines or the capacity to develop them. Strengthening capacity in these core components (CC4, CC6, CC7) would lead to adequate implementation of IPC guidelines and structures to reduce the burden of infections.

### Limitations

One of the limitations could be that some sections of the questionnaires administered may be perceived as compromising by some facilities. This may have adversely affected whether an objective response was obtained to some of the questions. Anonymizing the data obtained from the study facilities was done to enable objectivity in answering the questions.^46^ In the case of the IPCAF, its complexity and numerous footnotes and explanations, although thorough, may also leave room for misinterpretation and false reporting. An in-depth analysis of the responses to individual questions and answering patterns of the IPCAF questionnaire showed that responses were very varied, and thus beyond the scope of this project to describe them in detail here. Therefore, all the results could not be shown in this article due to the sheer amount of data the IPCAF collects and its complexity. The use of only three facilities also limits the generalizability of our results and conclusions within and beyond Ghana. The study also captured the input of two individuals at H1 and an individual each at H2 and H3, who although considered to be experts and therefore able to provide accurate answers, may have provided limited perspective and an increased potential for bias.

These limitations notwithstanding, this study has shown that healthcare facilities of different levels of care/services can utilize the WHO tools used in this study for objective AMS and IPC assessments. The study also highlights the importance of assessments of AMS and IPC programmes in diverse institutions in low-resource settings for quality improvement and patient safety.

### Conclusions and recommendations

The questionnaire used identified elements missing in each facility to support the establishment of functional AMS programmes. A nationwide survey of hospitals with this instrument may show similarities and patterns in the gaps identified across the country. The evidence generated provides guidance regarding interventions for AMS in the facilities assessed. The potential for improving antibiotic use and preventing or controlling infections is great considering the gaps identified in the AMS capacity assessment and the IPCAF to meet core elements needed to institute and sustain ASPs.^47^

All the facilities assessed had capacity to institute AMS programmes. The study revealed important gaps in relation to infrastructure, policy and practice, as well as monitoring and evaluation that need to be improved for optimal AMS.

Regarding IPC, it was observed that the facilities conformed to varying extents with the eight core components of practice guidelines recommended by WHO. All the facilities need to create and/or strengthen multidisciplinary teams such as the Drug and Therapeutic Committees (DTCs) to enable effective implementation of the guidelines for optimal antimicrobial therapy and IPC.

There is also the need to scale up AMR and antimicrobial consumption surveillance for robust data to support ASPs and effective implementation of IPC guidelines as part of sustainable efforts for AMR containment in Ghana. The findings from this work will be communicated to the study hospitals to support the institution and implementation of sustainable AMS programmes embedded with IPC to improve infectious disease prevention and therapy, and containment of the emergence and spread of AMR.

## Supplementary Material

dlac034_Supplementary_Data

## Supplementary data

The hospital questionnaire is available as Supplementary data at *JAC-AMR* Online.

## References

[1] Davies J . Where have All the Antibiotics Gone? Can J Infect Dis Med Microbiol 2006; 17: 287–90. 18382641 10.1155/2006/707296 PMC2095086

[2] Bebell LM , Muiru AN. Antibiotic use and emerging resistance: How can resource-limited countries turn the tide? Glob Heart 2014; 9: 347–58. 25667187 10.1016/j.gheart.2014.08.009 PMC4369554

[3] Ayukekbong JA , Ntemgwa M, Atabe AN. The threat of antimicrobial resistance in developing countries: Causes and control strategies. Antimicrob Resist Infect Control 2017; 6: 47. 28515903 10.1186/s13756-017-0208-x PMC5433038

[4] Brink AJ , Messina AP, Feldman C et al Antimicrobial stewardship across 47 South African hospitals: an implementation study. Lancet Infect Dis 2016; 16: 1017–25. 27312577 10.1016/S1473-3099(16)30012-3

[5] Dyar OJ , Huttner B, Schouten J et al What is antimicrobial stewardship? Clin Microbiol Infect 2017; 23: 793–8. 28882725 10.1016/j.cmi.2017.08.026

[6] Doron S , Davidson LE. Antimicrobial stewardship. Mayo Clin Proc 2011; 86: 1113–23. 22033257 10.4065/mcp.2011.0358 PMC3203003

[7] Pulcini C . Antibiotic stewardship: update and perspectives. Clin Microbiol Infect 2017; 23: 791–2. 28882723 10.1016/j.cmi.2017.08.020

[8] World Health Organization . Report on the Burden of Endemic Health Care-Associated Infection Worldwide. 2011. https://apps.who.int/iris/bitstream/handle/10665/80135/9789241501507_eng.pdf.

[9] Amponsah OKO , Buabeng KO, Owusu-Ofori A, et al Point prevalence survey of antibiotic consumption across three hospitals in Ghana. JAC-Antimicrob Resist 2021; 3: dlab008. 34223086 10.1093/jacamr/dlab008 PMC8210176

[10] Haque M , Sartelli M, McKimm J et al Health care-associated infections – An overview. Infect Drug Resist 2018; 11: 2321–3. 30532565 10.2147/IDR.S177247 PMC6245375

[11] Gilbert GL , Kerridge I. Hospital Infection Prevention and Control (IPC) and Antimicrobial Stewardship (AMS): Dual Strategies to Reduce Antibiotic Resistance (ABR) in Hospitals. In: Jamrozik E, Selgelid M, eds. Ethics and Drug Resistance: Collective Responsibility for Global Public Health. Public Health Ethics Analysis, Vol. 5. Springer, 2020.

[12] Byarugaba DK . Antimicrobial resistance in developing countries and responsible risk factors. Int J Antimicrob Agents 2004; 24: 105–10. 15288307 10.1016/j.ijantimicag.2004.02.015

[13] Founou RC , Founou LL, Essack SY. Clinical and economic impact of antibiotic resistance in developing countries: A systematic review and meta-analysis. PLoS One 2017; 12: e0189621. 29267306 10.1371/journal.pone.0189621 PMC5739407

[14] Ministry of Health, Ministry of Food and Agriculture, Ministry of Environment, Science, Technology and Innovation . National Action Plan (NAP) for Antimicrobial Use and Resistance in Ghana. 2017. https://www.moh.gov.gh/wp-content/uploads/2018/04/NAP_FINAL_PDF_A4_19.03.2018-SIGNED-1.pdf.

[15] World Health Organization . Interim practical manual: supporting national implementation of the WHO guidelines on core components of infection prevention and control programmes. 2017. https://apps.who.int/iris/handle/10665/330073.

[16] Hansen S , Zingg W, Ahmad R et al Organization of infection control in European hospitals. J Hosp Infect 2015; 91: 338–45. 26542950 10.1016/j.jhin.2015.07.011

[17] Struelens MJ , Wagner D, Bruce J et al Status of infection control policies and organisation in European hospitals, 2001: The ARPAC study. Clin Microbiol Infect 2006; 12: 729–37. 16842567 10.1111/j.1469-0691.2006.01462.x

[18] World Health Organization . Improving infection prevention and control at the health facility: interim practical manual supporting implementation of the WHO guidelines on core components of infection prevention and control programmes. 2018. https://apps.who.int/iris/handle/10665/279788.

[19] Oppong TB , Amponsem-Boateng C, Kyere EKD et al Infection prevention and control preparedness level and associated determinants in 56 acute healthcare facilities in Ghana. Infect Drug Resist 2020; 13: 4263–71. 33262620 10.2147/IDR.S273851 PMC7699444

[20] Yevutsey SK , Buabeng KO, Aikins M et al Situational analysis of antibiotic use and resistance in Ghana: Policy and regulation. BMC Public Health 2017; 17: 896–902. 29169340 10.1186/s12889-017-4910-7 PMC5701378

[21] Gyansa-Lutterodt M . Antibiotic resistance in Ghana. Lancet Infect Dis 2013; 13: 1006–7. 24252479 10.1016/S1473-3099(13)70196-8

[22] World Health Organization . WHO Methodology for Point Prevalence Survey on Antibiotic Use in Hospitals. 2018. https://www.who.int/publications/i/item/WHO-EMP-IAU-2018.01.

[23] Ejisu Municipal-Ghana Districts: A repository of all Local Assemblies in Ghana. https://www.ghanadistricts.com/Home/District/18.

[24] Asante Akim North-Ghana Districts: A repository of all Local Assemblies in Ghana. https://www.ghanadistricts.com/Home/District/9.

[25] KMA Ghana Districts: A repository of all Local Assemblies in Ghana. http://ghanadistricts.com/Home/District/20.

[26] World Health Organization . Infection Prevention and Control Assessment Framework at the Facility Level. 2018. https://www.who.int/infection-prevention/tools/core-components/IPCAF-facility.PDF.

[27] Vanderbilt Software – REDCap. REDCap - Research Electronic Data Capture. doi:10.1111/j.1365-2583.2009.00888.x ER.

[28] StataCorp . Stata Statistical Software: Release 14. 2015.

[29] Chukwu EE , Oshun PO, Osuolale KA et al Antimicrobial stewardship programmes in healthcare facilities in Lagos State, Nigeria: a needs assessment. J Glob Antimicrob Resist 2021; 25: 162–70. 33812050 10.1016/j.jgar.2021.02.034

[30] Fadare JO , Ogunleye O, Iliyasu G et al Status of antimicrobial stewardship programmes in Nigerian tertiary healthcare facilities: findings and implications. J Glob Antimicrob Resist 2019; 17: 132–6. 30557686 10.1016/j.jgar.2018.11.025

[31] Baker SN , Acquisto NM, Ashley ED et al Pharmacist-managed antimicrobial stewardship program for patients discharged from the emergency department. J Pharm Pract 2012; 25: 190–4. 22095578 10.1177/0897190011420160 PMC3533486

[32] Haque A , Hussain K, Ibrahim R et al Impact of pharmacist-led antibiotic stewardship program in a PICU of low/middle-income country. BMJ Open Qual 2018; 7: e000180. 10.1136/bmjoq-2017-000180 PMC5759741 29333498

[33] Zingg W , Storr J, Park BJ et al Implementation research for the prevention of antimicrobial resistance and healthcare-associated infections; 2017 Geneva infection prevention and control (IPC)-think tank (part 1). Antimicrob Resist Infect Control 2019; 8: 1–9. 31161034 10.1186/s13756-019-0527-1 PMC6540528

[34] Hsia Y , Lee BR, Versporten A et al Use of the WHO Access, Watch, and Reserve classification to define patterns of hospital antibiotic use (AWaRe): an analysis of paediatric survey data from 56 countries. Lancet Glob Health 2019; 7: e861–71. 31200888 10.1016/S2214-109X(19)30071-3

[35] WHO . The 2019 WHO AWaRe classification of antibiotics for evaluation and monitoring of use. 2019. https://apps.who.int/iris/handle/10665/327957.

[36] van der Sandt N , Schellack N, Mabope LA et al Surgical antimicrobial prophylaxis among pediatric patients in South Africa comparing two healthcare settings. Pediatr Infect Dis J 2019; 38: 122–6. 29677085 10.1097/INF.0000000000002072

[37] Ierano C , Manski-Nankervis J-A, James R et al Surgical antimicrobial prophylaxis. Aust Prescr 2017; 40: 225–9. 29377021 10.18773/austprescr.2017.073 PMC5768598

[38] Commission on Safety and Quality in Health Care . Antimicrobial prescribing practice in Australian hospitals. 2016. https://www.safetyandquality.gov.au/publications-and-resources/resource-library/antimicrobial-prescribing-practice-australian-hospitals-results-2016-hospital-national-antimicrobial-prescribing-survey.

[39] Saleem Z , Hassali MA, Versporten A et al A multicenter point prevalence survey of antibiotic use in Punjab, Pakistan: findings and implications. Expert Rev Anti Infect Ther 2019; 17: 285–93. 30755077 10.1080/14787210.2019.1581063

[40] Al Matar M , Enani M, Binsaleh G et al Point prevalence survey of antibiotic use in 26 Saudi hospitals in 2016. J Infect Public Health 2019; 12: 77–82. 30270148 10.1016/j.jiph.2018.09.003

[41] Labi AK , Obeng-Nkrumah N, Nartey ET et al Antibiotic use in a tertiary healthcare facility in Ghana: A point prevalence survey. Antimicrob Resist Infect Control 2018; 7: 15. 29423190 10.1186/s13756-018-0299-z PMC5787245

[42] Versporten A , Zarb P, Caniaux I et al Antimicrobial consumption and resistance in adult hospital inpatients in 53 countries: results of an internet-based global point prevalence survey. Lancet Glob Health 2018; 6: e619–29. 29681513 10.1016/S2214-109X(18)30186-4

[43] Kritsotakis EI , Astrinaki E, Messaritaki A et al Implementation of multimodal infection control and hand hygiene strategies in acute-care hospitals in Greece: A cross-sectional benchmarking survey. Am J Infect Control 2018; 46: 1097–103. 29778434 10.1016/j.ajic.2018.04.217

[44] Storr J , Twyman A, Zingg W et al Core components for effective infection prevention and control programmes: New WHO evidence-based recommendations. Antimicrob Resist Infect Control 2017; 6: 1–18. 10.1186/s13756-016-0149-9 PMC5223492 28078082

[45] Aghdassi SJS , Hansen S, Bischoff P et al A national survey on the implementation of key infection prevention and control structures in German hospitals: Results from 736 hospitals conducting the WHO Infection Prevention and Control Assessment Framework (IPCAF). Antimicrob Resist Infect Control 2019; 8: 73. 31080588 10.1186/s13756-019-0532-4 PMC6505265

[46] Ong AD , Weiss DJ. The impact of anonymity on responses to sensitive questions. J Appl Soc Psychol 2000; 30: 1691–708.

[47] US CDC . Core Elements of Hospital Antibiotic Stewardship Programs. https://www.cdc.gov/antibiotic-use/core-elements/hospital.html. 10.1093/cid/ciu542 PMC6521960 25261548

