# Supplementary material for: Antimicrobial stewardship capacity and infection prevention and control assessment of three health facilities in the Ashanti Region of Ghana
Source: JAC Antimicrob Resist. 2022 Apr 9;4(2):dlac034. doi: 10.1093/jacamr/dlac034 (PMC8994196; doi:10.1093/jacamr/dlac034)

**Supplementary data**

Please note that the questionnaire below is an extract from the *WHO Methodology for Point Prevalence Surveys* (<https://www.who.int/publications/i/item/WHO-EMP-IAU-2018.01>), see page 74, and has not been altered.

Appendix 2: WHO HOSPITAL QUESTIONNAIRE (ANNEX XII)


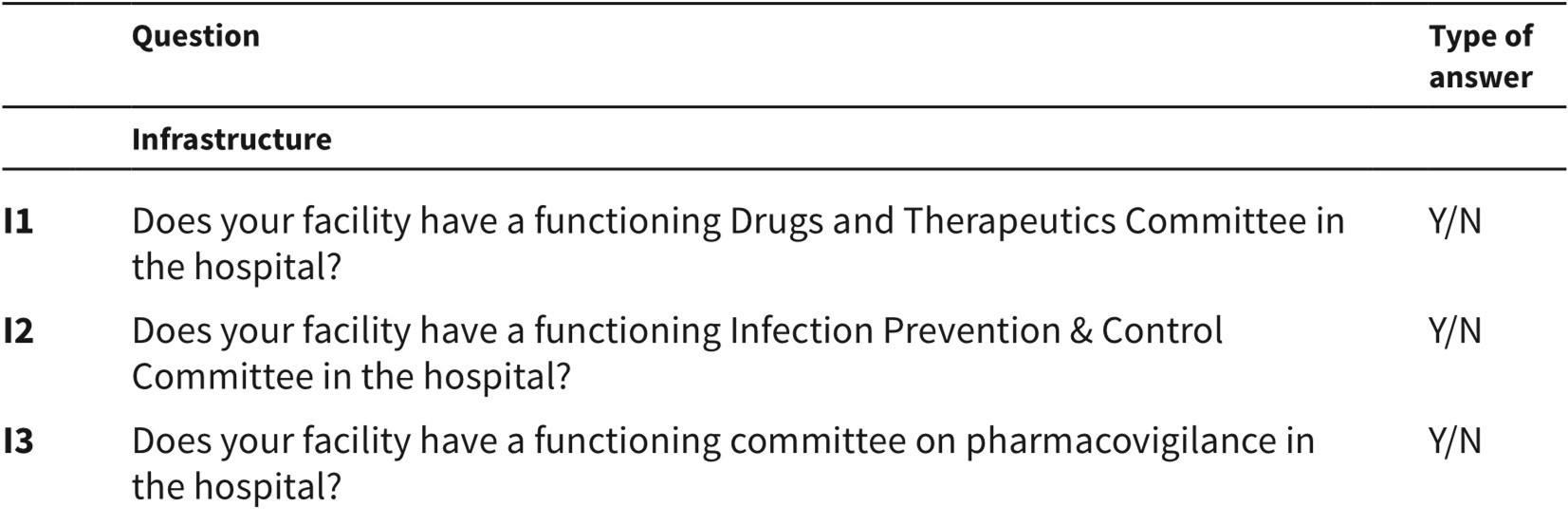

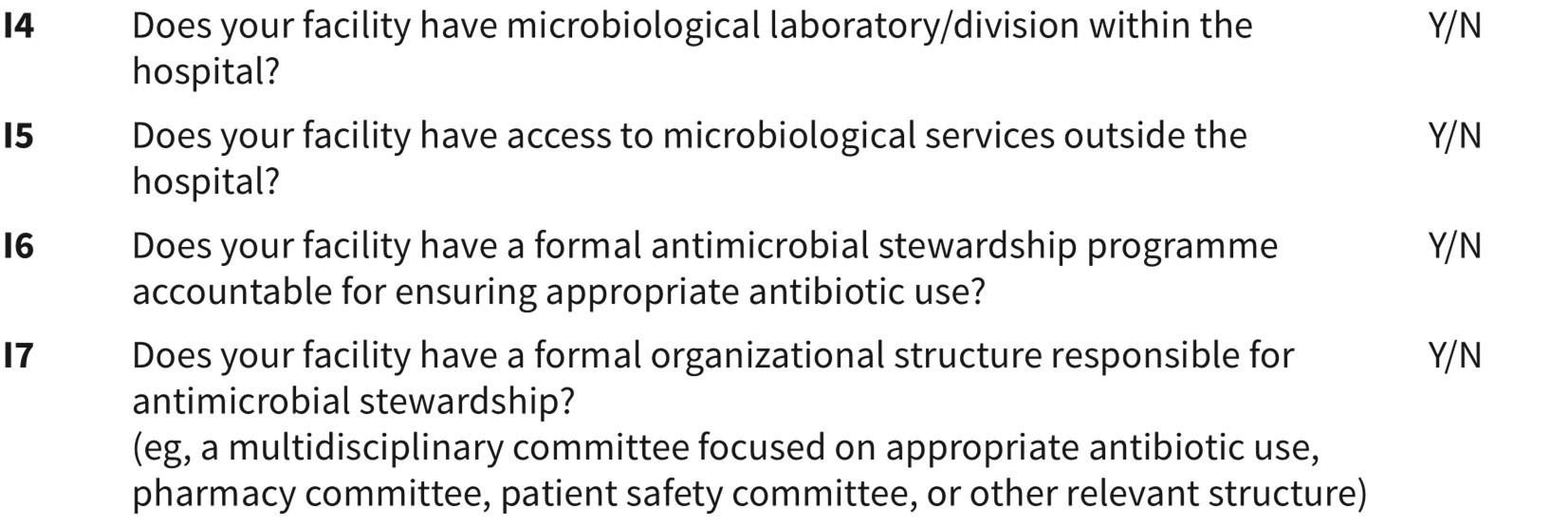

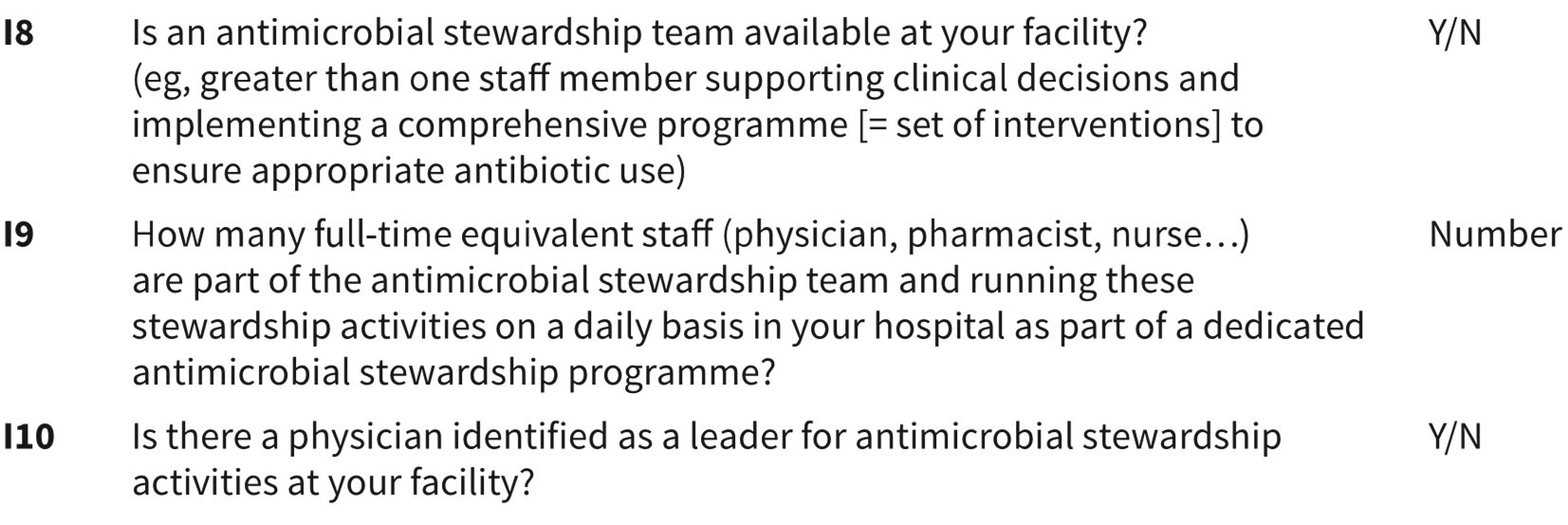

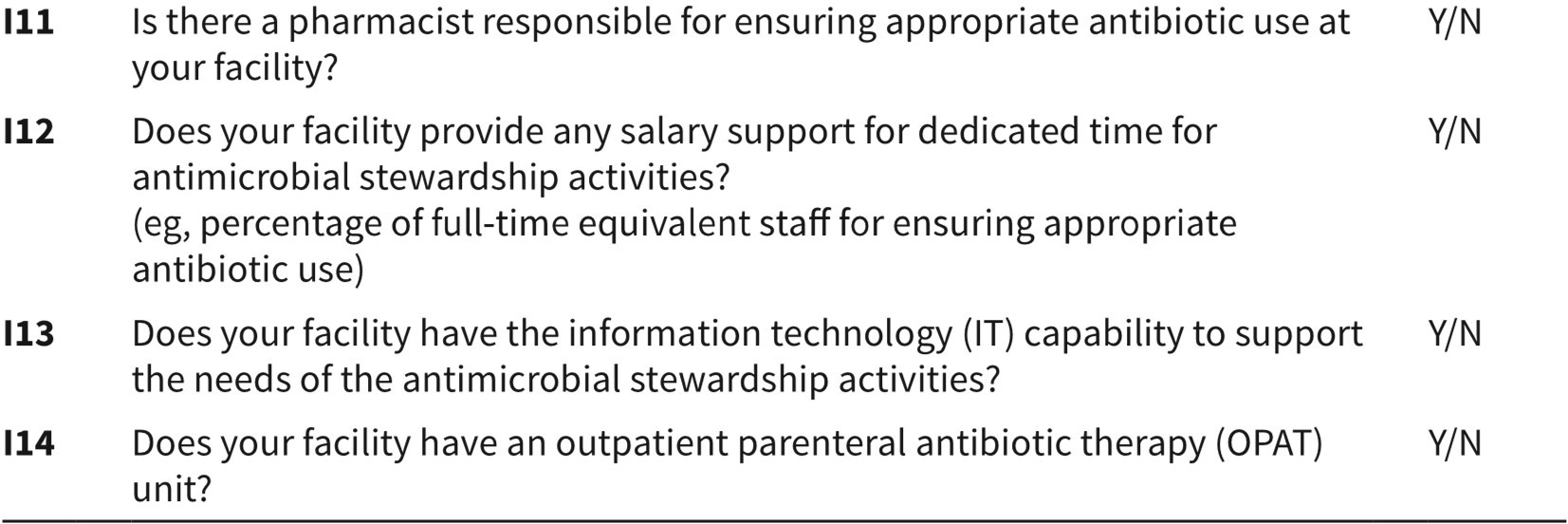


16


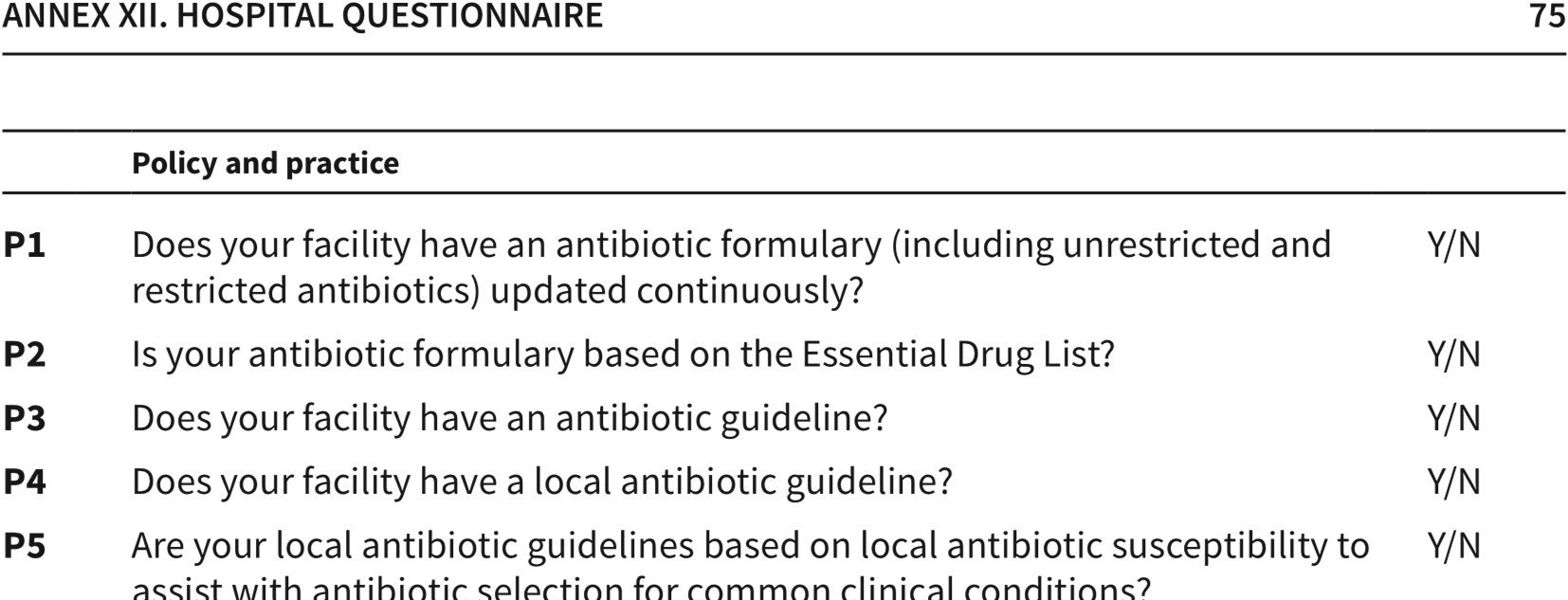

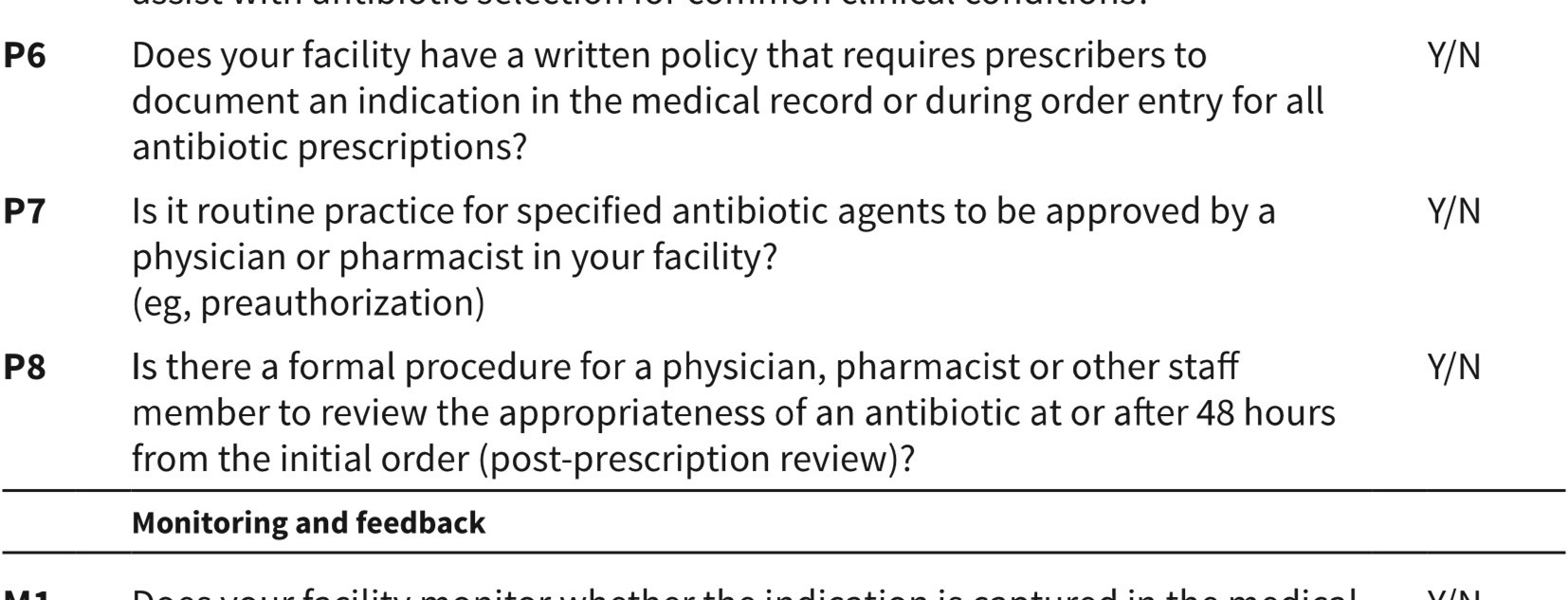

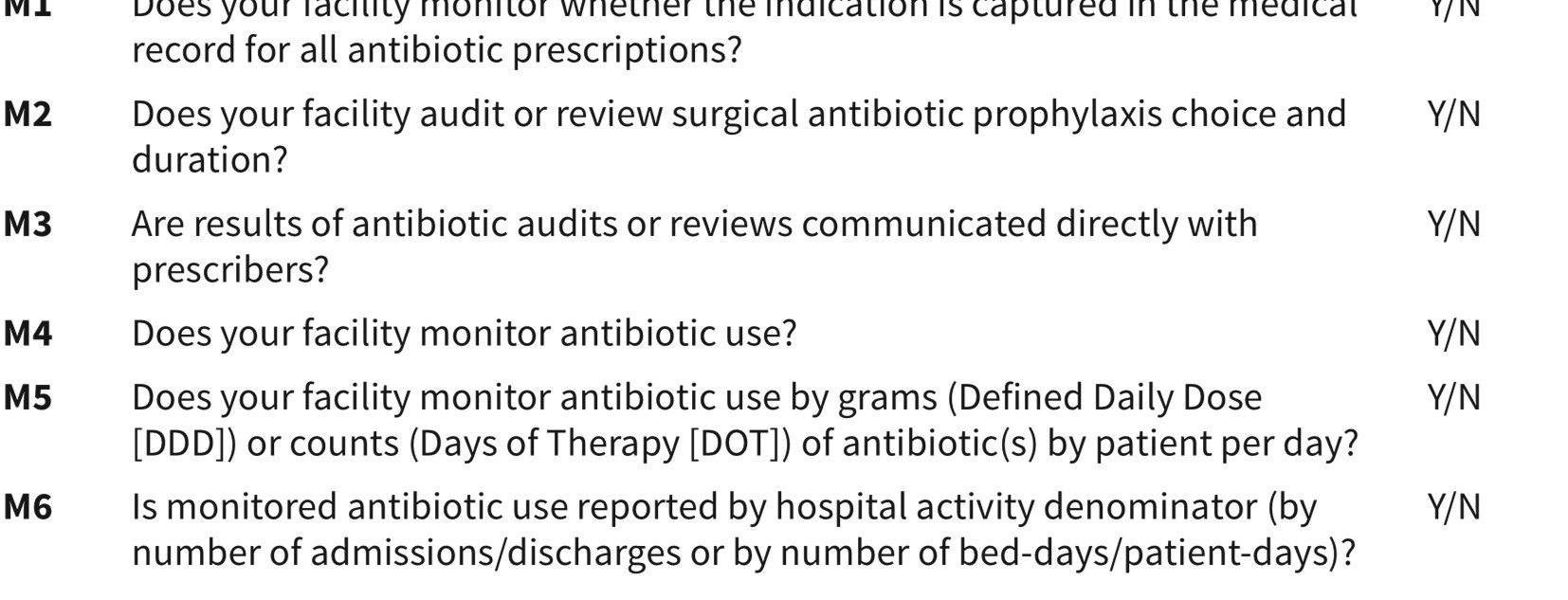

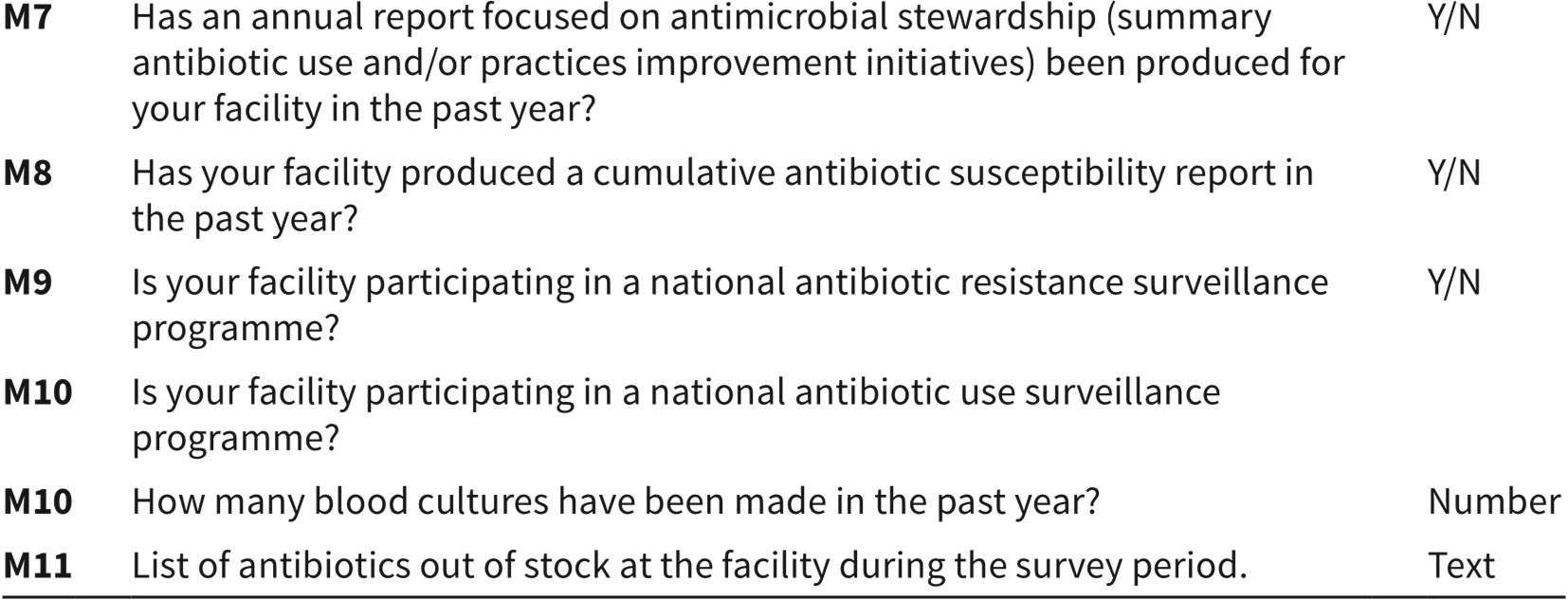

Supplement: dlac034_Supplementary_Data [file dlac034_supplementary_data.docx]
